# Congenitally Corrected Transposition of the Great Arteries in a Septuagenarian from the Developing Country of Pakistan

**DOI:** 10.7759/cureus.2737

**Published:** 2018-06-05

**Authors:** Hunaina Shahab, Salima Ashiqali, Mehnaz Atiq

**Affiliations:** 1 Medicine, Aga Khan University Hospital, Karachi, PAK; 2 Cardiopulmonary Department, Aga Khan University Hospital, Karachi, PAK; 3 Department of Paediatrics & Child Health, Aga Khan University Hospital, Karachi, PAK

**Keywords:** congenital cardiac disease, transposition of great arteries, echocardiography, electrocardiography

## Abstract

Congenitally corrected transposition of the great arteries (CCTGA) is a rare congenital cardiac defect with atrioventricular and ventriculoarterial discordance which leads to heart failure and limits patients’ lifespan. We report the case of a 70-year-old lady, from a poor province in Pakistan, who presented for the first time with palpitations and was diagnosed to have CCTGA. She had moderate pulmonic valve stenosis which was protective against heart failure. She had six children all born via spontaneous vertex delivery in her local village. This case exemplifies the fact that pulmonic stenosis is favourable for patients with CCTGA. In a country where the average life expectancy of females is only 68 years, the survival of our patient with CCTGA beyond the average lifespan is indeed interesting.

## Introduction

von Rokistansky in 1875 was the first to describe a congenital cardiac anomaly known as the congenitally corrected transposition of the great arteries (CCTGA) [1]. CCTGA is a defect whereby the right atrium is connected to the left ventricle, supplying blood to the pulmonary artery, whereas the left atrium is connected to the right ventricle, supplying blood to the aorta [2]. The disorder still allows deoxygenated blood to be carried by the pulmonary artery to the lungs and oxygenated blood through the aorta to the systemic circulation [3].

It is a rare defect that makes up about 0.5 to 1% of congenital cardiac diseases [2,4]. The defect has a slight male predominance and there is about a 5% risk of recurrence of congenital cardiac diseases in families with CCTGA [3,5]. The life expectancy of CCTGA patients with associated defects is limited [4]. Those patients with isolated CCTGA have reduced life spans due to the development of congestive right-sided heart failure by the fourth or fifth decade of life [6]. We describe the case of a 70-year-old lady from Pakistan, who presented for the first time in the clinic with palpitations and was found to have CCTGA with associated pulmonic stenosis.

## Case presentation

A 70-year-old lady, belonging to a poor village in the province of Balochistan, presented to the cardiology clinics at Aga Khan University Hospital, Karachi, Pakistan with complaints of palpitations. She was married with six children, all born in her village via spontaneous vertex delivery with no complications. She had previously been diagnosed with hypertension by a local general practitioner and had been taking bisoprolol 5 mg once a day for elevated blood pressures. She had started experiencing palpitations for the past one month. She did not complain of any chest pain or syncope but mentioned having dyspnea on climbing two flights of stairs for the past 25 years. Her family history was significant for diabetes and hypertension only.

On physical examination, her heart rate was 72 beats per minute, blood pressure was 148/76 mmHg and oxygen saturation was 96% on room air. There was neither clubbing nor cyanosis. On cardiac auscultation, she was found to have grade 3/6 crescendo-decrescendo murmur at the pulmonic area radiating to the left suprascapular region and left lower sternal border. Chest auscultation revealed normal vesicular breathing and the abdomen was soft, non-tender with no visceromegaly.

**Figure 1:**
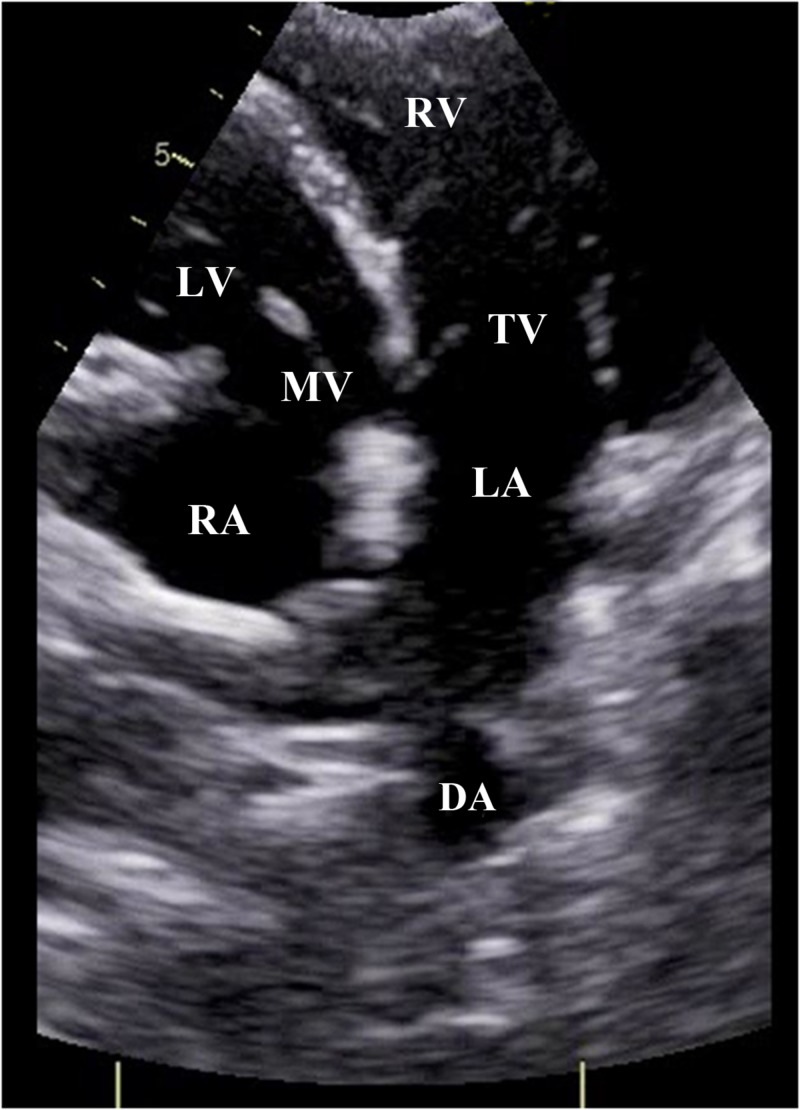
Echocardiogram. Apical four chamber view showing right atrium (RA) connected to left ventricle (LV) through mitral valve (MV). Left atrium (LA) is connected to right ventricle (RV) through tricuspid valve (TV). Descending aorta (DA) is also seen.

An echocardiogram was done which showed that the visceroatrial situs was solitus with levocardia. The interatrial septum was thin and aneurysmal but had no defect. Pulmonary venous connections were normal. Single right-sided superior vena cava and inferior vena cava drained into the right atrium. The atrio-ventricular connection was discordant. Tricuspid valve was normal on the left side. There was moderate tricuspid regurgitation with maximum pressure gradient of 50 mmHg. Mitral valve was normal on the right side with no mitral regurgitation. Ventricular inversion was noted with the systemic ventricle (right ventricular morphology) being dilated with mild hypertrophy. Right ventricular (RV) systolic function was mildly reduced with a tricuspid annular plane systolic excursion (TAPSE) of 12 mm. There was no left ventricular (LV) hypertrophy and LV systolic function was normal. The inter-ventricular septum was intact as shown in Figure 1.

**Figure 2:**
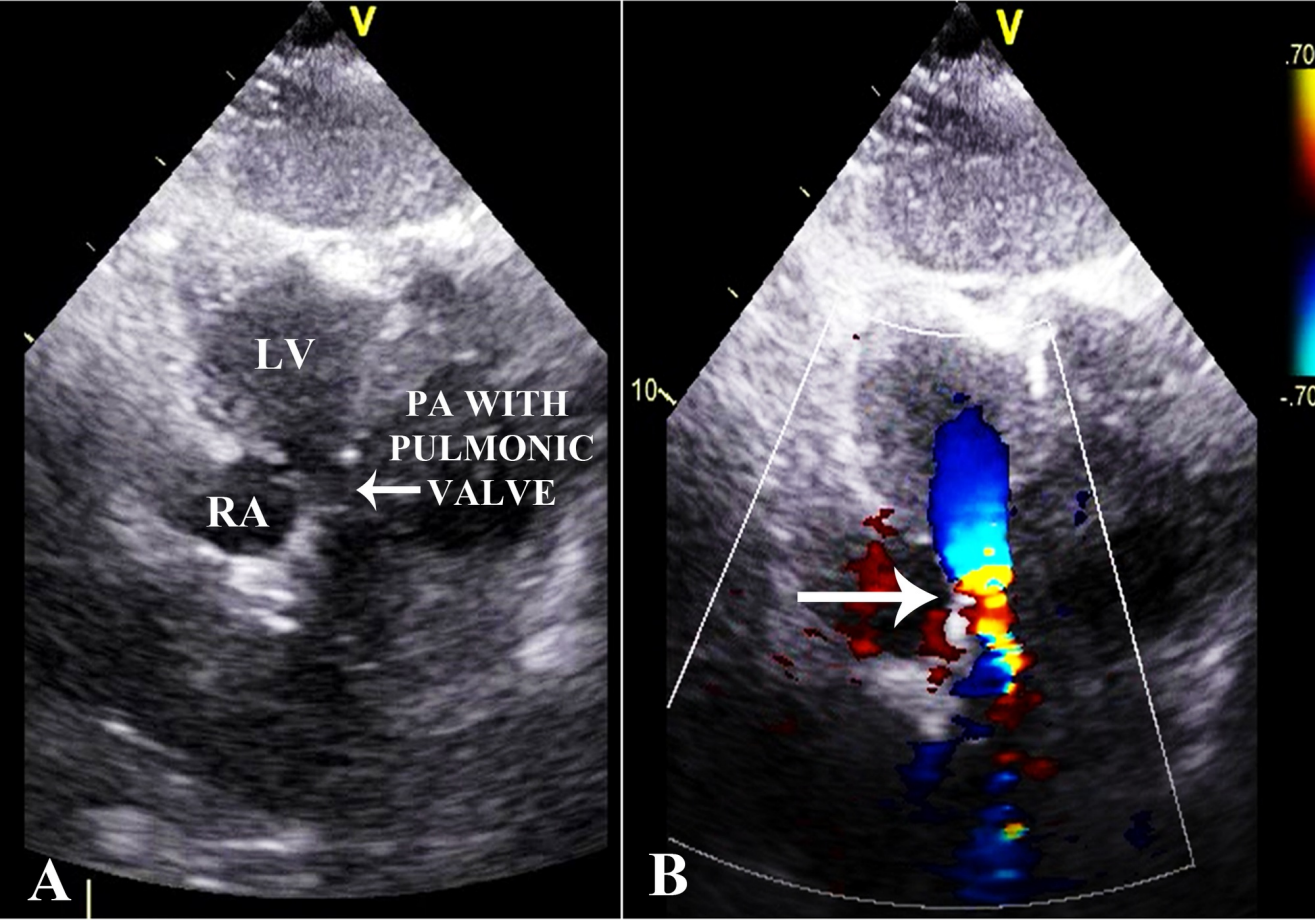
Echocardiogram. (A) Modified view showing pulmonary artery (PA) along with pulmonic valve arising from the left ventricle (LV). (B) Modified view with color Doppler applied at the level of the pulmonic valve. Forward turbulence can be seen denoting stenosis at the level of the pulmonic valve (arrow).

**Figure 3:**
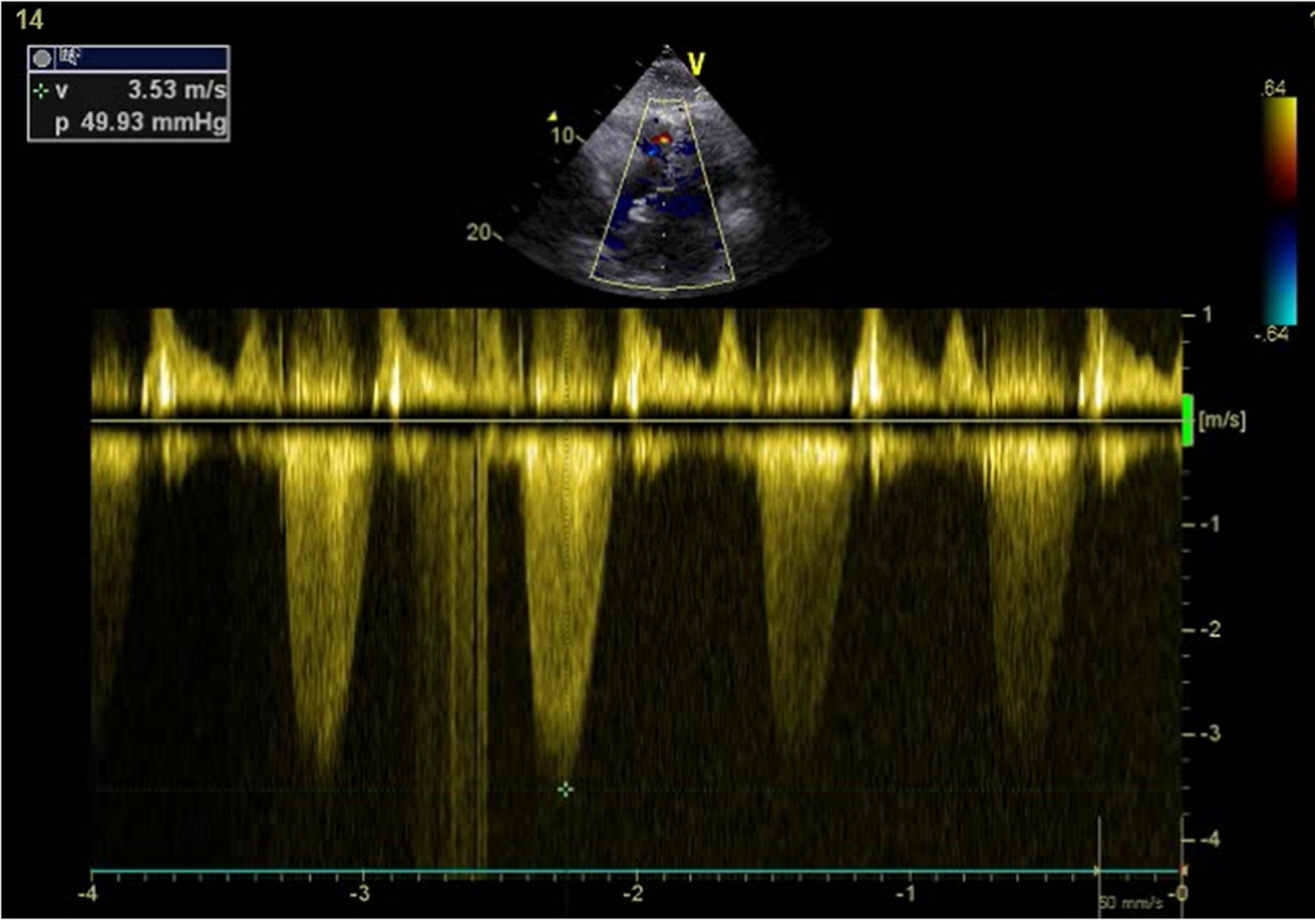
Echocardiogram. Continuous wave Doppler applied at the level of the pulmonic valve showing maximum pressure gradient of 50 mmHg denoting moderate pulmonary valve stenosis.

Ventriculo-arterial connection was discordant. Pulmonary artery arose from LV as shown in Figure 2A. Aorta originated from the RV, anterior and to the left of the pulmonary artery. The aortic valve was normal with no stenosis or regurgitation. Pulmonary valve was thickened, domed and dysplastic with moderate pulmonary stenosis and a maximum pressure gradient of 50 mmHg as shown in Figures 2B, 3.

 

**Figure 4:**
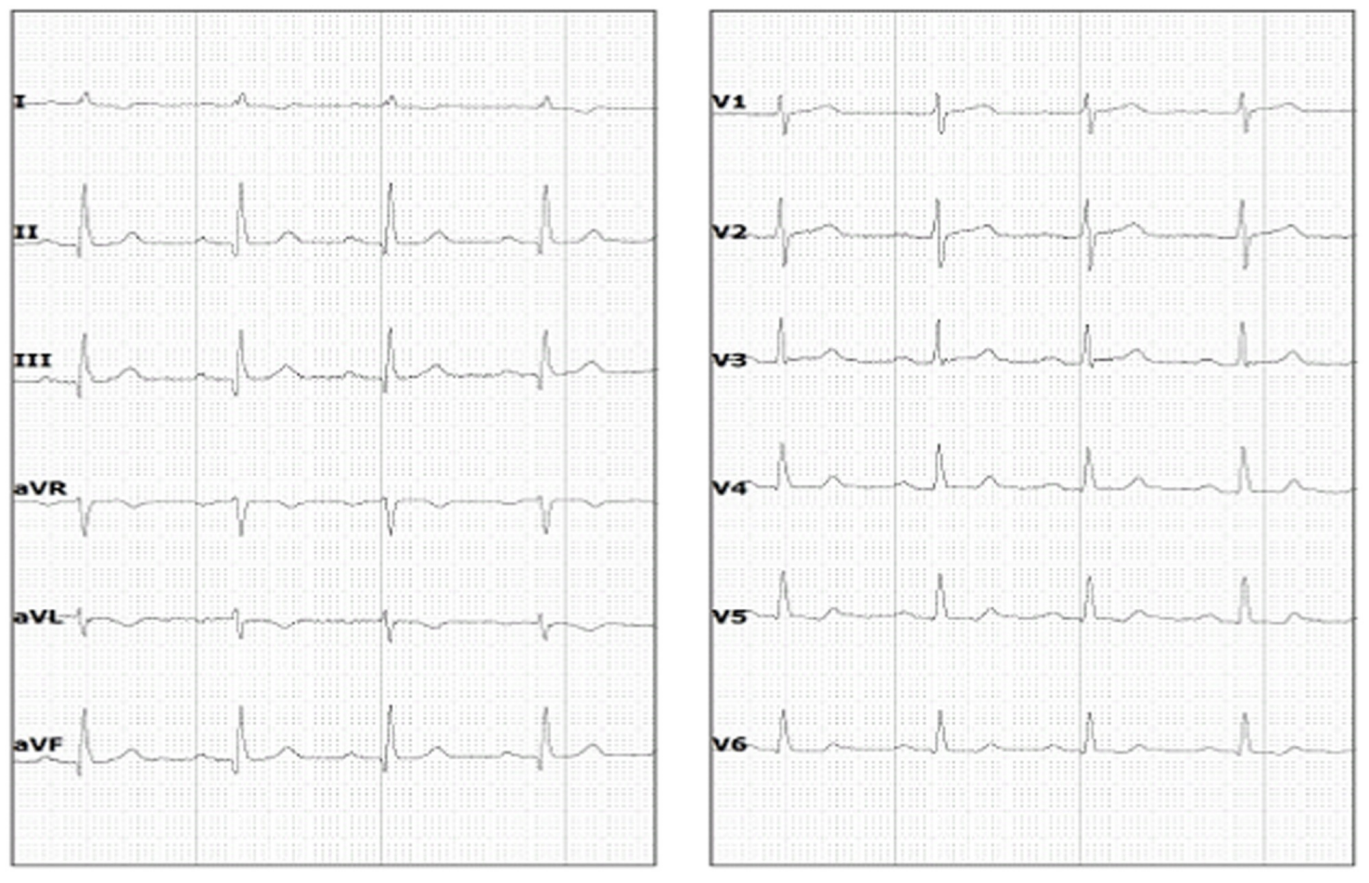
Electrocardiogram. Twelve lead electrocardiogram of the patient showing normal sinus rhythm with normal axis and small Q waves in leads II, III and AVF.

There was no pulmonary regurgitation. Post-stenotic dilatation was identified. Pulmonary valve annulus was severely hypoplastic and measured 9.6 mm (z score -5.4) with main pulmonary artery size being 17 mm (z score -1.4), right pulmonary artery size 14 mm (z score +0.2) and left pulmonary artery size 12 mm (z score +0.2). Neither aortic coarctation nor patent ductus arteriosus was seen. A 24 hour Holter monitor revealed no significant abnormalities. She remained in normal sinus rhythm throughout the Holter monitoring even during periods where she complained of palpitations. Her electrocardiogram (ECG) is shown in Figure 4.

It was decided to keep her on medical management. Perindopril was started and the previous beta-blocker was continued. On a two month follow-up, her symptoms had improved. It was planned to closely follow her in the outpatient clinics.

## Discussion

Up to 90% of patients with CCTGA are reported to have other cardiac anomalies like tricuspid or mitral valve abnormalities, pulmonary artery stenosis and ventricular septal defects [7], the latter two being the most common [2]. Symptomatology of these patients depends on the criticality of the associated anomalies and right ventricular function [8]. These patients are known to develop congestive heart failure as the right ventricle is subjected to both pressure and volume overload [9]. Since pulmonary outflow obstruction is naturally protective against heart failure in CCTGA patients, pulmonary artery banding shows favorable results [9]. This is due to an increased afterload on the left ventricle, which leads to reduced interventricular septal shift to the LV, resulting in reduced deformation of RV shape and function [10]. Hence, CCTGA patients who have associated ventricular septal defect and pulmonic stenosis have somewhat better outcomes [11]. In our case, the naturally occurring pulmonic stenosis protected our patient against congestive heart failure, helping her survive with minimal symptoms till the ripe old age of 70 years.

There have been multiple case reports of CCTGA being diagnosed in patients of older age [12-14]. Till date, the oldest reported patient with CCTGA is a 92-year-old woman from France [14]. To the best of our knowledge, this is the first reported case of CCTGA in a 70-year-old from the developing country of Pakistan. The average life expectancy of females in Pakistan is about 68 years [15]. Therefore, in a country with limited health care facilities, the survival of our patient with a congenital heart disease limiting life expectancy is indeed interesting. What is even more fascinating is that she successfully gave birth to six children via spontaneous vertex delivery in a remote village with minimal medical facilities. This shows that CCTGA patients even with associated cardiac defects can conceive and deliver uneventfully. This is similar to a study from the United Kingdom where 74% of the women with CCTGA had uncomplicated pregnancies [16]. Furthermore, conduction abnormalities are frequently associated with CCTGA [17] due to the anomalous location of the atrioventricular (AV) node and the AV bundle [7]. Therefore, we assessed our patient with 24 hour Holter monitoring which did not reveal any conduction defects. She remained in normal sinus rhythm even during the times when she felt palpitations.

## Conclusions

CCTGA is a rare congenital condition with the development of heart failure in mid-adulthood. Our case exemplifies the fact that pulmonic stenosis in CCTGA is protective against heart failure, contributing to a favourable prognosis of the defect in adults. Moreover, pregnancy can be carried out successfully in CCTGA patients with such associated anomalies.
